# Procalcitonin as a Predictor of Septic Knee Arthritis: A Retrospective Cohort Study

**DOI:** 10.5435/JAAOSGlobal-D-22-00261

**Published:** 2023-01-09

**Authors:** Kevin West, Hasan Almekdash, John Fisher, Alexis D. Rounds, Jefferson Murphree, Jordan Simpson

**Affiliations:** From the Department of Orthopaedic Surgery, (Dr. West, Fisher, Dr. Rounds, and Dr. Simpson); the Clinical Research Institute (Dr. Almekdash), Texas Tech University Health Science Center, Lubbock, TX; and the Department of Orthopaedics and Rehabilitation, University of Florida School of Medicine, Gainesville, FL (Dr. Murphree).

## Abstract

Differentiating septic arthritis from aseptic arthritis (AA) of the knee is difficult without arthrocentesis. Although procalcitonin (PCT) has shown diagnostic value in identifying bacterial infections, it has not been established as a reliable marker for identifying septic arthritis (SA). Recent studies have shown promise in the use of PCT as a useful systemic marker for identifying septic arthritis versus AA. This observational retrospective review compares PCT with routine inflammatory markers as a tool for differentiating septic arthritis versus AA in patients with acute, atraumatic knee pain.

**Methods:**

Fifty-three consecutive patients (24 SA, 29 AA) were retrospectively reviewed at one institution with concern for SA. SA was diagnosed based on a physical examination, laboratory markers, and arthrocentesis. Laboratory indices were compared between the septic arthritis and AA groups. Data analysis was conducted to define sensitivity and specificity. Receiver operator characteristic curve analysis and regression were conducted to determine the best marker for acute SA of the knee.

**Results:**

Using multiple logistic regression, bacteremia (OR 6.75 ± 5.75) was determined to be the greatest predictor of SA. On linear regression, concomitant bacteremia (coef 3.07 ± 0.87), SA (coef 2.18 ± 0.70), and the presence of pseudogout crystals (coef 1.80 ± 0.83) on microscopy predicted an increase in PCT. Using a PCT cutoff of 0.25 ng/mL yields a sensitivity of 91.7% and specificity of 55.2% for predicting SA; however, the ideal cutoff in our series was 0.32 ng/mL with a sensitivity of 79.2% and specificity of 72.4%. PCT was superior to the white blood cell count, erythrocyte sedimentation rate, and C-reactive protein in the area under the receiver-operating characteristic curve analysis.

**Discussion:**

Procalcitonin seems to be the most sensitive and specific systemic marker in differentiating septic from AA.

Early diagnosis and treatment of septic arthritis (SA) can help mitigate the joint destruction that can occur from prolonged infection. Currently, there is no single laboratory marker available to reliably diagnose SA. The current benchmark of arthrocentesis with cell count, Gram stain, and culture has a reported sensitivity of 50% to 75% and takes days for a definitive diagnosis.^1^ Although very specific, arthrocentesis is invasive in nature, aspiration can cause pain, yield dry taps, and poses a potential risk of iatrogenic infection.^2^ The latter of which is more apparent with an increasing prevalence of arthroplasty.^3,4^ These limitations have driven a search for alternative tools to rapidly and accurately diagnose SA.

Laboratory tests have become a focus in exploring alternative tools for diagnosing SA. Serum white blood cell (WBC) count, erythrocyte sedimentation rate (ESR), and C-reactive protein (CRP) are often used as predictors of SA. However, these markers individually, and as a whole, have low diagnostic value because of inadequate sensitivity and specificity.^5^ Since its discovery in 1975, the body of literature on serum procalcitonin (PCT) as a useful biomarker for bacterial infections has grown.^6^

Advantages of PCT include its rapid rise to detectable levels within 2 to 6 hours of initiation of acute infection,^7–9^ its specificity to bacterial infections without influence by other states of inflammation, such as viral or autoimmune processes,^7^ and the timeliness of results.^10^ This allows rapid detection of sepsis and reduces antibiotic exposure in nonbacterial infections.^8^

Translating the utility of PCT to benefit patients who present to the emergency department with suspected SA has proven to be a challenge. A number of studies have looked into the use of PCT for detecting SA.^7,11,12,13,14,15^ Although several meta-analyses in native and prosthetic joints have reported high sensitivities in the 95% range, the specificities remained low around 55%.^16,17^ However, these studies used a PCT cutoff value of 0.5 ng/ml^16^ or an inconsistent cutoff,^17^ which likely negatively affected the sensitivity level. Hugle et al^1^ proposed using a PCT cutoff of 0.25 ng/mL and reported increased sensitivity for detecting SA at 93% while maintaining a high specificity at 93%. The goal of this study was to further validate the sensitivity, specificity, and utility of PCT as a diagnostic marker for SA at this cutoff. This study hypothesized that a serum PCT cutoff of 0.25 ng/mL would be superior to WBC, ESR, and CRP at discerning SA from aseptic arthritis (AA).

## Methods

This is a retrospective review at a single institution of adult patients presenting to our emergency department between January 2017 and October 2018 with atraumatic knee pain and concern for SA. This project was reviewed and approved by the institutional review board. A priori power analysis was conducted with an enrollment goal of 50 patients. Patients with involvement of joints other than the knee, those on antibiotics within the preceding week of the encounter, those who underwent any surgery within 5 days preceding the encounter, and patients with incomplete workup as described in the next paragraph were excluded. This resulted in the exclusion of nine patients. A total of 53 knees in 51 patients were included for final analysis. The mean age was 61 ± 17.4 years, and 35 patients (68%) were male. Patient demographics are presented in Table 1.

**Table 1:** Patient Demographics

Demographic	Total	Aseptic	Septic	*P* value
	n = 51	n = 27 (52.9%)	n = 24 (47.1%)	
Age, years^a^	61.4 ± 17.4	63.6 ± 19.6	58.9 ± 14.7	0.343
Male	35 (68.6%)	21 (77.8%)	14 (58.3%)	0.135
BMI^a^	30.8 ± 6.5	29.5 ± 6.0	32.2 ± 6.9	0.135
Comorbidities^b^	3.2 ± 2.0	2.8 ± 1.9	3.4 ± 2.1	0.333
Chronic obstructive pulmonary disease	2 (3.9%)	2 (7.4%)	0 (0.0%)	0.170
Hyperlipidemia	15 (29.4%)	10 (37.0%)	5 (20.8%)	0.205
Osteoarthritis	7 (13.7%)	5 (18.5%)	2 (8.3%)	0.255
Rheumatoid arthritis	1 (2.0%)	0 (0.0%)	1 (4.2%)	0.284
Hypothyroidism	8 (15.7%)	5 (18.5%)	3 (12.5%)	0.555
Cerebrovascular disease	3 (5.9%)	2 (7.4%)	1 (4.2%)	0.623
Peptic ulcer disease	3 (5.9%)	2 (7.4%)	1 (4.2%)	0.623
Hypertension	31 (60.8%)	17 (63.0%)	14 (58.3%)	0.735
Heart arrhythmia	5 (9.8%)	3 (11.1%)	2 (8.3%)	0.739
Coronary artery disease	8 (15.7%)	4 (14.8%)	4 (16.7%)	0.856
Chronic kidney disease	9 (17.7%)	5 (18.5%)	4 (16.7%)	0.863
Peripheral vascular disease	4 (7.8%)	2 (7.4%)	2 (8.3%)	0.902
Liver disease	4 (7.8%)	2 (7.4%)	2 (8.3%)	0.902
Dementia	2 (3.9%)	1 (3.7%)	1 (4.2%)	0.932
Cancer	2 (3.9%)	1 (3.7%)	1 (4.2%)	0.932
Diabetes	19 (37.3%)	10 (37.0%)	9 (37.5%)	0.973

a Age, body mass index, and the number of comorbidities were normally distributed; therefore, they are reported as mean ± SD

b Only comorbidities considered in the Charlson Comorbidity Index affecting at least one patient or those affecting five or more patients in the total cohort are reported here

### Diagnostic Workup

Patients were evaluated with a physical examination and laboratory studies including complete blood cell count, ESR, CRP, and PCT. Arthrocentesis was conducted to obtain Gram stain, culture, cell count, and polarization microscopy of synovial fluid. SA was suspected based on physical examination, systemic indices, and arthrocentesis results. A positive diagnosis of SA was confirmed with a positive culture. Diagnosis of periprosthetic joint infection was made based on the 2018 Musculoskeletal Infection Society criteria.^18^ Crystal arthropathy was identified on microscopy using polarized birefringence and stratified into gout with monosodium urate crystals or pseudogout with calcium pyrophosphate dihydrate crystals. Alternative sources of infection were investigated through the use of chest radiograph, urinalysis, blood culture, and review of documentation. If no abnormalities were found after a complete workup, the final diagnosis was knee pain of unknown etiology.

### Statistical Analysis

All statistical analyses were conducted in Stata Multi-Processing edition 13 (StataCorp). The Shapiro-Wilk test was used to test for normality of distribution of continuous variables. Patients were stratified by the presence or absence of SA. Continuous demographic variables were normally distributed and, therefore, compared using the Student t-test (reported as mean ± SD) and Pearson chi square test for categorical data. All laboratory test results except ESR were non-normally distributed; therefore, the Mann-Whitney U test was used to compare all laboratory data (reported as median ± interquartile range). Bivariate followed by multivariate logistic regression was used by purposeful selection to determine ORs for risk of SA. Purposeful selection was done again for bivariate and multivariate linear regression to determine coefficients for increased PCT. We completed ROC analysis and measured the area under the curve (AUC) to determine the ideal cutoff for each biomarker because they relate to identifying septic arthritis and AA. All data with *P* values < 0.05 were considered significant.

## Results

Of these 51 patients, 24 patients (47.1%) were diagnosed with SA, and 27 patients (52.9%) (one patient with bilateral knee pain and one patient presenting with knee pain on two separate visits) with AA of varying etiologies. No statistically significant differences were observed between the SA and AA patients in age, sex, body mass index, or comorbidities (Table 1).

### Patient Characteristics

All knee characteristics are presented in Table 2. There were 25 knees (47.2%) with the presence of crystals on microscopy consisting of 15 gout (28.3%) and 10 pseudogout (18.9%). The presence of crystal arthropathy and types of crystals were not significantly different between the SA and AA groups (Table 2). There were 25 patients (47.2%) who had some other infectious process occurring outside the knee joint. These were broken down into other infections about the knee and infectious processes unrelated to the knee, which were not significantly different between SA and AA knees. Of the 8 knees (15.1%) with other infections about the knee, four (7.6%) had cellulitis, one (1.9%) had septic bursitis, and three (5.7%) had a superficial abscess. A total of 20 knees (37.7%) had infectious processes unrelated to the knee including five urinary tract infections (9.4%); three cases (5.7%) of pneumonia; two epidural abscesses (3.8%); and one (1.9%) of each fungemia, empyema, pyelonephritis, and colitis. The number of positive blood cultures [11 (26.2%)] trended toward increased SA (*P* = 0.052), and bacteremia [10 (18.9%)] was significantly higher in the SA cohort (8 [33.3% vs. 2 6.9%], *P* = 0.014). The number of infections, types of infections, and locations of the infections were not different between the two groups. Our cohort also included 10 knees (18.9%, *P* < 0.001) with underlying total knee arthroplasty, which were all found to be septic (*P* < 0.001) with positive cultures.

**Table 2:** Knee Characteristics

Characteristics	Total	Aseptic	Septic	*P* value
	n = 53	n = 29 (54.7%)	n = 24 (45.3%)	
Right knee	32 (60.4%)	15 (51.7%)	17 (70.8%)	0.157
Total knee arthroplasty	10 (18.9%)	0 (0.0%)	10 (41.7%)	<0.001
Crystals	25 (47.2%)	16 (55.2%)	9 (37.5%)	0.200
Gout	15 (28.3%)	10 (34.5%)	5 (20.8%)	0.272
Pseudogout	10 (18.9%)	6 (20.7%)	4 (16.7%)	0.709
Other infectious process	25 (47.2%)	12 (41.4%)	13 (54.2%)	0.353
One	20 (37.7%)	9 (31.0%)	11 (45.8%)	
Two	4 (7.6%)	2 (6.9%)	2 (8.3%)	
Three	1 (1.9%)	1 (3.5%)	0 (0.0%)	
Other knee Infection	8 (15.1%)	4 (13.8%)	4 (16.7%)	0.771
Cellulitis	4 (7.6%)	1 (3.5%)	3 (12.5%)	0.214
Bursitis	1 (1.9%)	1 (3.5%)	0 (0.0%)	0.358
Abscess	3 (5.7%)	2 (6.9%)	1 (4.17%)	0.669
Non–knee infection	20 (37.7%)	10 (34.5%)	10 (41.7%)	0.591
Bacteremia	10 (18.9%)	2 (6.9%)	8 (33.3%)	0.014
Positive blood culture	11 (26.2%)	3 (13.6%)	8 (40.0%)	0.052
Urinary tract infection	5 (9.4%)	4 (13.8%)	1 (4.2%)	0.233
Fungemia	1 (1.9%)	1 (3.5%)	0 (0.0%)	0.358
Pyelonephritis	1 (1.9%)	1 (3.5%)	0 (0.0%)	0.358
Colitis	1 (1.9%)	1 (3.5%)	0 (0.0%)	0.358
Pneumonia	3 (5.7%)	2 (6.9%)	1 (4.2%)	0.669
Epidural abscess	2 (3.8%)	1 (3.5%)	1 (4.2%)	0.891

### Aseptic Arthritis Cohort

There were 29 knees (54.7%) in 27 patients (52.9%) included in the AA cohort. The final etiology of the acute knee pain was determined to be crystal arthropathy in 16 knees (55.2%) consisting of 10 gout (34.5%) and six pseudogout (20.7%). One patient had microscopy-confirmed gout in bilateral knees. When considering knees without crystal arthropathy, the final cause of the knee pain was determined to be osteoarthritis in three patients (10.3%); superficial abscess in two patients (6.9%); and then one patient (3.4%) of each cellulitis, septic bursitis, and posttraumatic arthritis. One patient presented twice for recurrent hemarthrosis in the same knee approximately 2 months apart. The final three patients (10.3%) were determined to have knee pain of unknown etiology.

Total twelve knees (41.4%) with AA were found to have an active infection outside the knee joint, and three knees (10.3%) had positive blood cultures. Of these patients, one (3.5%) had Methicillin resistant staphylococcus aureus (MRSA) bacteremia with MRSA septic knee bursitis, one (3.5%) had MSSA bacteremia with a superficial abscess overlying the knee and an epidural abscess, and another (3.5%) had Candida spp. fungemia. Two patients (6.9%) had pneumonia, one (3.5%) of whom had an empyema. Three patients (10.3%) had a urinary tract infection, and one patient (3.5%) had pyelonephritis. One additional patient (3.5%) had Clostridium difficile colitis, and another (3.5%) had pyelonephritis. Total three patients (10.3%) in the AA cohort had two sources of infection. Other than the two patients presented previously, the other patient had pneumonia and a urinary tract infection. Six patients (20.7%) with AA had crystal arthropathy with a concomitant infection (3 gout, three pseudogout).

### Septic Arthritis Cohort

Of the 24 patients (45.3%) with SA, 14 (58.3%) were native knees and 10 (41.7%) were prosthetic. Microorganisms were isolated in 100% of the SA cultures, which included 16 (66.6%) with Staphylococcus aureus (seven [29.2%] MRSA), four (16.6%) Streptococcus spp. (1 [4.2%] group A, two [8.3%] group B, one [4.2%] mitis), and one (4.2%) of each *Staphylococcus epidermidis*, *Pasteurella multocida*, *Bacteroides fragilis*, and Cutibacterium acnes.

Thirteen patients (54.2%) with SA had at least one additional infectious process. There were 11 patients (45.8%) with one and two patients (8.3%) with two additional infectious processes. Four (16.7%) of these infections were about the knee, including three (12.5%) with overlying cellulitis and one (4.2%) with a superficial abscess overlying the knee. There were 10 patients (41.7%) with an additional infectious process unrelated to the knee including eight (40.0%) with bacteremia, one (4.2%) with an epidural abscess and pneumonia, and one (4.2%) had a urinary tract infection. One SA patient (4.2%) had simultaneous bacteremia and cellulitis. Nine SA patients (37.5%) had a concomitant diagnosis of microscopy-confirmed crystal arthropathy including five gout (20.8%) and four pseudogout (16.7%). There were five patients (20.8%) with SA who had concurrent bacteremia and crystal arthropathy (two gout [8.3%], three pseudogout [12.5%]).

### Laboratory Markers

Overall, inflammatory markers were higher in the SA group (Table 3). The WBC count (12.6 vs. 10.8, *P* = 0.313) and ESR (76 vs. 59, *P* = 0.103) trended toward being higher in the SA group but was not significant. SA patients demonstrated significantly higher CRP levels (27.0 vs. 8.6, *P* = 0.021) and cell count (68,700 vs. 13,100, *P* < 0.001) than their counterparts. Similarly, patients with SA had significantly higher levels of PCT than those with AA (0.74 vs. 0.20, *P* < 0.001). Inflammatory markers in the AA cohort all decreased after the exclusion of other infectious processes with the exception of WBC. The ESR became significantly higher in the SA cohort after this exclusion (76 [51 to 100] vs. 47 [37 to 70], *P* = 0.030). Of note, PCT levels trended toward being higher in patients with pseudogout [1.31 (0.25 to 3.05) vs. 0.29 (0.18 to 0.86), *P* = 0.111] and when infections of any kind (including SA) were excluded (0.23 [0.13 to 0.42] vs. 0.15 [0.06 to 0.20], *P* = 0.083).

**Table 3:** Comparison of Laboratory Markers Between Groups

Laboratory Marker^a^	Total	Aseptic	Septic	*P* value
	n = 53	n = 29 (54.7%)	n = 24 (45.3%)	
WBC	11.9 (9.6-15.3)	10.8 (9.6-14.6)	12.6 (9.9-16.9)	0.313
ESR	66.5 (44.5-93.5)	59.0 (37.0-76.0)	76.0 (51.0-100.0)	0.103
CRP	18.5 (6.2-28.8)	8.6 (4.3-18.8)	27.0 (19.9-31.5)	0.021
Cell count	27.5 (7.5-65.9)	13.1 (4.3-24.4)	68.7 (30.9-132.0)	<0.001
PCT	0.34 (0.18-1.44)	0.20 (0.11-0.37)	0.74 (0.36-3.00)	<0.001
After exclusion of patients with other infectious processes	n = 41	n = 17	n = 24	
WBC	11.9 (9.9-16.2)	10.8 (9.9-15.3)	12.6 (9.9-16.9)	0.672
ESR	59.5 (44.5-92.5)	47.0 (37.0-70.0)	76.0 (51.0-100.0)	0.030
CRP	19.9 (6.2-29.3)	6.7 (4.3-18)	27.0 (19.9-31.5)	0.001
Cell count	34.3 (8.3-75.5)	10.5 (5.3-24.4)	68.7 (30.9-132.0)	<0.001
PCT	0.38 (0.18-1.74)	0.18 (0.10-0.23)	0.74 (0.36-1.03)	<0.001

CRP = C-reactive protein, ESR = erythrocyte sedimentation rate, PCT = procalcitonin, WBC = white blood cell

a Reported as median (interquartile range)

### Regression

A bivariate regression to identify possible predictors of SA was used (Table 4). Purposeful selection was used to build a multivariate model initially using all variables with *P* < 0.200. Only bacteremia predicted SA in the final model with an OR of 6.75 ± 5.75 (95% confidence interval (CI), 1.27 to 35.80, *P* = 0.013). Bivariate linear regression was used to determine variables that influence PCT (Table 5). The final multivariate model included the presence of SA (coefficient 2.18 ± 0.70, 95% CI, 0.78 to 3.58, *P* = 0.003), bacteremia (coefficient 3.07 ± 0.87, 95% CI, 1.32 to 4.83, *P* = 0.001), and pseudogout (coefficient 1.80 ± 0.83, 95% CI, 0.14 to 3.46, *P* = 0.034).

**Table 4:** Bivariate Logistic Regression for Risk of Septic Arthritis

Characteristics^a^	OR^c^	95% Confidence Interval	*P* value
Age	0.99 ± 0.02	0.96-1.02	0.423
Male^b^	0.37 ± 0.23	0.11-1.23	0.097
Number of comorbidities	0.87 ± 0.13	0.65-1.15	0.320
BMI^b^	1.07 ± 0.05	0.98-1.17	0.137
Hyperlipidemia^b^	0.43 ± 0.27	0.12-1.49	0.173
Osteoarthritis	0.44 ± 0.39	0.08-2.48	0.332
Other infection	1.67 ± 0.93	0.56-4.99	0.353
Bacteremia^b^	6.75 ± 5.75	1.27-35.80	0.013
Cellulitis	4.00 ± 4.76	0.39-41.23	0.209
Crystal arthropathy^c^	0.49 ± 0.27	0.16-1.47	0.198
Pseudogout	0.53 ± 0.36	0.14-2.02	0.340
Gout	0.69 ± 0.45	0.19-2.48	0.568

a Total knee arthroplasty perfectly predicted SA and was, therefore, not included.

b Included in initial multiple logistic regression, only bacteremia was significant in the final model.

c Reported as OR ± standard error.

**Table 5:** Bivariate and Multivariate Linear Regression for Correlation With Procalcitonin

	Bivariate			Multivariate		
Characteristics	Coefficient^a^	95% Confidence Interval	*P* value	Coefficient^a^	95% Confidence Interval	*P* value
Age	−0.01 ± 0.02	−0.05 to 0.03	0.678			
Male	−0.80 ± 0.82	−2.45 to 0.86	0.337			
BMI	0.00 ± 0.06	−0.11 to 0.12	0.948			
Osteoarthritis	−0.36 ± 1.13	−2.62 to 1.91	0.753			
Hyperlipidemia	−0.54 ± 0.83	−2.2 to 1.12	0.519			
Septic arthritis	1.98 ± 0.72	0.55-3.42	0.008	2.18 ± 0.70	0.78-3.58	0.003
Other Infection^b^	1.13 ± 0.75	−0.37 to 2.63	0.138			
Bacteremia^b^	3.57 ± 0.84	1.89-5.26	<0.001	3.07 ± 0.87	1.32-4.83	0.001
Cellulitis	−0.47 ± 1.44	−3.37 to 2.42	0.744			
Crystal Arthropathy^b^	1.04 ± 0.75	−0.47 to 2.54	0.174			
Gout	0.01 ± 0.89	−1.77-1.79	0.991			
Pseudogout^b^	1.46 ± 0.89	−0.32 to 3.25	0.106	1.80 ± 0.83	0.14-3.46	0.034

a Reported as coefficient ± standard error.

b Variables included in initial multiple linear regression. SA, bacteremia, and pseudogout were significant in the final model.

### ROC Analysis

**Figure 1:**
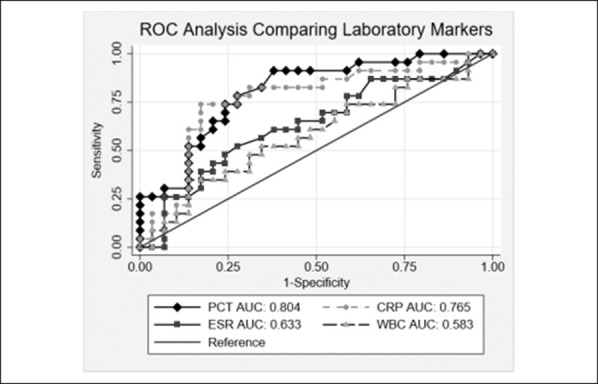
Graph showing ROC analysis of laboratory values for all patients

On Receiver Operating Characteristic Curve (ROC) analysis, the AUC for PCT was 0.804 (95% CI, 0.683 to 0.922), which was significantly better than WBC (0.583, 95% CI, 0.423 to 0.739, *P* = 0.021) and not significantly different from ESR (0.633, 95% CI, 0.684 to 0.924, *P* = 0.092), CRP (0.765, 95% CI, 0.633 to 0.906, *P* = 0.6747), or cell count (0.865, 95% CI, 0.759 to0.971, *P* = 0.371) (Figure 1).

Using a PCT cutoff of 0.25 ng/mL, sensitivity and specificity were 91.7% and 55.2%, respectively. The cutoff points with the highest combined sensitivity and specificity were calculated for PCT (0.32 ng/mL, sensitivity 79.2%, specificity 72.4%), CRP (21.1 mg/dL, sensitivity 75.0%, specificity 82.8%), and ESR (no optimal cutoff).

**Figure 2:**
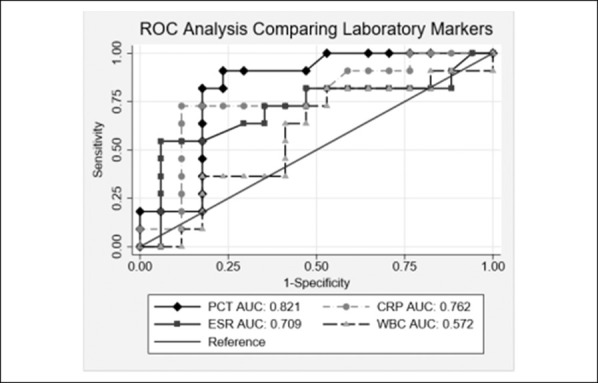
Graph showing ROC analysis of laboratory values excluding those with another infection

After excluding patients with an infectious process other than SA, AUCs improved for each laboratory marker (Figure 2). With this exclusion, sensitivity and specificity improved to 90.9% and 76.5%, respectively, with an ideal cutoff of more than 0.25 ng/mL. Without excluding other infections, 100% sensitivity for PCT was reached at 0.09 ng/mL. The negative predictive value at 0.10 ng/mL is 87.5%. With exclusion, 100% sensitivity was reached at 0.18 ng/mL.

## Discussion

Over the past two decades, the support for the use of PCT as a useful systemic marker for bacterial infections has grown. During homeostasis, serum PCT levels are minimal (<0.05 ng/mL).^7^ Exposure to bacterial infection and lipopolysaccharide upregulates PCT production creating a detectable elevation of PCT blood plasma concentration.^6^ During an infection, the concentration of PCT in serum rises rapidly between 2 and 6 hours and peaks within 6 to 24 hours during an acute infection. This small latency from the initiation of an infection to detectable levels of PCT in the blood makes PCT a particularly useful marker during the initial stages of infection.^7–9^

The difficult clinical scenario of differentiating between septic and aseptic causes of knee pain has led to notable exploration into different biomarkers. Although helpful, the routine biomarkers WBC, ESR, and CRP can be elevated in patients with chronic inflammatory diseases, obesity, and smoking.^19,20,21,22^ Currently, the benchmark to diagnose SA remains arthrocentesis.^1^ With the ever-increasing prevalence of arthroplasty,^3,4^ an accurate diagnosis using systemic biomarkers is appealing. Furthermore, the low sensitivity of Gram stain and delay in culture results have led to research into other systemic biomarkers such as PCT, TNF-α, IL-6, D-dimer, and others with varying success.^1,8,11,16,17,20,23–25^

Multiple studies have previously investigated the value of PCT in the SA diagnostic algorithm.^1,16,17^ The meta-analysis by Zhao et al^16^ and the articles presented in it^7,11,12,13,14,15,26^ used a cutoff of 0.5 ng/ml. As such, PCT was found effective at ruling in SA with a specificity of 95%, but the sensitivity of 54% rendered this test inadequate at ruling out SA. Using ROC analysis, Hugle et al^1^ found that a cutoff of 0.25 ng/mL rather than 0.5 ng/mL increased the sensitivity of PCT to 93% while maintaining specificity at 75%. The specificity improved to 93% while maintaining a sensitivity of 93% when other infective processes were accounted for.

Compared with the findings of Hugle et al, our study yielded a similar sensitivity (92% vs. 93%), but a lower specificity (55% vs. 73%). On ROC analysis, our ideal cutoff point was found to be 0.32 ng/mL compared with 0.25 ng/mL in the study by Hugle, which resulted in decreased sensitivity (79%) and improved specificity (72%) relative to our initial findings. After excluding other infectious processes that likely increase the PCT level, our ideal cutoff point was the same as that reported by Hugle et al. This cutoff of 0.25 ng/mL yielded a sensitivity of 91% and specificity of 77% in our study compared with 93% and 93%, respectively, in that by Hugle. Interestingly, this study found that an NPV of 100% occurs at a PCT of < 0.09 ng/mL, which is very similar to that first noted by Hugle et al. (<0.10 ng/mL). The NPV at the level reported by Hugle in our study was 87.5%. Overall, this study supports the notion that when PCT < 0.10 ng/mL, the likelihood of a patient having SA is very low and nonseptic causes for arthritis can be confidently explored. Overall, both studies found that PCT outperformed CRP and WBC on AUC in ROC analysis. Of note, Hugle et al did not study ESR or cell count. Our study found that AUC for PCT was not inferior to cell count on ROC analysis, which is commonly used to guide early treatment while waiting on Gram stain and culture.

One unexpected finding that was discovered during our data analysis was that pseudogout was a positive predictor of increasing PCT on multivariate linear regression analysis. We found this reported once previously in a case report,^27^ but have not seen it otherwise discussed in the literature. However, no significant differences were noted in initial comparisons of the median with the Mann-Whitney U test, suggesting that there are likely confounders. Given the intent to identify PCT as a marker to differentiate SA from AA, an elevation with pseudogout does potentially cloud the clinical picture.

Our study supports a PCT cutoff point of 0.25 ng/mL in discerning SA from AA of the knee. If these findings can be further validated, there is potential to reduce invasive arthrocentesis and antibiotic administration in patients without bacterial arthritis. This can be particularly useful in patients with preexisting autoimmune or inflammatory conditions that damper the utility of nonspecific inflammatory biomarkers. Unfortunately, there were not enough patients with autoimmune diseases or immunosuppressants to find useful results. Obtaining PCT levels in these patients could facilitate quicker and more informed treatment decisions because the test may reliably rule out SA if the serum PCT levels are below 0.25 ng/mL.

By including patients with total knee arthroplasty, this study explored a potentially valuable tool in evaluating PJI. This has been previously reviewed by Yoon et al;^17^ however, several studies included in the meta-analysis excluded patients with preexisting inflammatory conditions. A larger cohort that includes arthroplasty patients and patients with preexisting inflammatory conditions is needed either to determine the utility of PCT for diagnosis or to help determine whether a knee should be aspirated.

This study has several limitations. First, our data were collected in a retrospective manner which is subject to selection bias; however, this was mitigated by collecting consecutive patients. Second, this is a sampling of 53 patients at a single institution, which may not extrapolate to a larger patient cohort. Third, our study only involves the knee joint and does not include other less commonly affected joints such as the hip, shoulder, wrist, and ankle. Attempts to extrapolate the data to other joints should be done with caution pending larger, more inclusive studies. Finally, native and prosthetic joint infections were included in our study, yet the two situations may warrant different diagnostic approaches.

When looking globally at identifying infections within the knee joint, our study indicates that PCT works well. Like the study by Hugle et al, our study suggests that PCT is effective at identifying septic arthritis from AA and is superior to many of the routine systemic biomarkers when a threshold of 0.25 ng/mL is used. Furthermore, the results of this study support that the likelihood of SA is exceedingly low with a PCT level of < 0.1 ng/mL. The relationship between elevated PCT levels and pseudogout is not understood, but this study implies that PCT may help specifically identify pseudogout among other inflammatory processes. We think PCT is a valuable tool in differentiating septic arthritis from AA and, if further validated, may help guide treatment and prevent unnecessary arthrocentesis.

## Acknowledgments

TTUHSC Clinical Research Institute.
